# High-throughput cognitive assessment using BrainTest.org: examining cognitive control in a family cohort

**DOI:** 10.1002/brb3.158

**Published:** 2013-08-02

**Authors:** Fred W Sabb, Gerhard Hellemann, Deanna Lau, Jessica R Vanderlan, Heather J Cohen, Robert M Bilder, James T McCracken

**Affiliations:** Semel Institute for Neuroscience and Human Behavior Brain Research Institute David Geffen School of Medicine, University of CaliforniaLos Angeles, California

**Keywords:** Cognitive control, inattention, spatial working memory, symptoms, WWW

## Abstract

**Introduction** Understanding the relationship between brain and complex latent behavioral constructs like cognitive control will require an inordinate amount of data. Internet-based methods can rapidly and efficiently refine behavioral measures in very large samples that are needed for genetics and behavioral research. Cognitive control is a multifactorial latent construct that is considered to be an endophenotype in numerous neuropsychiatric disorders, including attention deficit/hyperactivity disorder (ADHD). While previous studies have demonstrated high correlations between Web- and lab-based scores, skepticism remains for its broad implementation. **Methods** Here, we promote a different approach by characterizing a completely Web-recruited and tested community family sample on measures of cognitive control. We examine the prevalence of attention deficit symptoms in an online community sample of adolescents, demonstrate familial correlations in cognitive control measures, and use construct validation techniques to validate our high-throughput assessment approach. **Results** A total of 1214 participants performed Web-based tests of cognitive control with over 200 parent–child pairs analyzed as part of the primary study aims. The data show a wide range of “subclinical” symptomatology in a web community sample of adolescents that supports a dimensional view of attention and also provide preliminary narrow-sense heritability estimates for commonly used working memory and response inhibition tests. **Conclusions** Finally, we show strong face and construct validity for these measures of cognitive control that generally exceeds the evidence required of new lab-based measures. We discuss these results and how broad implementation of this platform may allow us to uncover important brain–behavior relationships quickly and efficiently.

## Introduction

In order to understand the neurobiology of complex behavioral processes like cognitive control (Miller and Cohen 2001), the ability to exert control over one's thoughts and actions, we need to validate high-throughput methods, including unsupervised testing of large numbers of participants in parallel via the Internet. There are numerous benefits to high-throughput behavioral assessment, from achieving sample sizes needed for testing genetic associations, to reducing the logistical hurdles in testing complex familial designs, and refinement of latent behavioral constructs through efficient iterative measurement development. Here, we use this approach to tackle several challenging problems in behavioral research, including efficient examination of a large sample and testing of both parents and offspring, to determine the symptom profile of adolescents in a Web-community sample, and provide initial insights into the heritability of frequently used cognitive tests. Further, we demonstrate the validity of this entirely Web-based design by using traditional construct validity analytic approaches to help overcome lingering skepticism about web assessment.

There are significant gaps in our understanding of not only the neurobiology of cognitive control but even the very definition and expression of the construct. Improved understanding of the component processes attributed to cognitive control through iterative construct and measurement refinement can lead to more tractable studies of the neural and genetic bases of behavior, which in turn may even have clinical implications by helping to elucidate the underlying causes of neuropsychiatric disease. A number of reviews report that working memory and response inhibition are components of cognitive control (Pennington 1997; Sabb et al. 2008). These constructs are also correlated with highly heritable neuropsychiatric diseases including schizophrenia and attention deficit hyperactivity disorder (ADHD), demonstrating that examination of basic psychological processes in healthy community individuals can impact knowledge about major mental illness. Yet, outside of extensive work by Plomin and colleagues on genetic linkage for “*g*” (e.g., Plomin and Spinath 2002), there are few genetic association studies of cognitive constructs (but see Need et al. 2009). Further, the scant reproducible evidence from psychiatric genetics for categorical disorders (produced in part by noise in the construct definition) should provide an even stronger role for psychological research. The challenge, however, in collecting enough cognitive test data using validated measures to conduct well-powered genetic linkage or association studies remains a barrier.

Using a high-throughput unsupervised platform like the World Wide Web can help to overcome this problem. Although a number of studies have demonstrated strong validity with respect to Web-based testing, broad adoption has continued to elude the field. The web offers virtually limitless sample size, the ability to collect complex family structures in an extremely cost effective manner, and the speed to test and refine constructs and measurements in days or weeks instead of months or years. A number of studies have conducted traditional comparisons of scores for Web- and lab-based cognitive assessment, showing correlations at the ceiling of lab test–retest numbers (e.g., Silverstein et al. 2007; Haworth et al. 2009; Germine et al. 2012). We propose that construct validation procedures are more appropriate for demonstration of the utility and validity of Web-based assessment. Such methods have been used successfully before (Krantz and Dalal 2000; McGraw et al. 2000; Silverstein et al. 2007). Our goal was to build on these previous studies and again specifically highlight the importance of construct development and validation in studying cognitive control via the Web.

Here, we present our Web-based platform to measure cognitive constructs and show strong construct validity using classical test-development tools. We report prevalence of attention symptoms using an adapted scale in our Web-based community cohort, relationships between symptoms and cognitive variables, and suggest heritability of psychological measures. These data begin to build a large normative sample of Web-based responses. We discuss the putative inertial bias in the broad adoption of web testing and suggest how our evidence can help overcome this, toward a path of high-throughput assessment necessary to understand the neurobiology of complex psychological processes.

## Methods

### Participants

A total of 1214 volunteers from the community underwent informed consent procedures online (approved by UCLA IRB). Parents under 55 years with a child between the ages of 9–17 were eligible. Many adult individuals, however, performed the measures for fun without recruiting children. Recruitment was done through measures to those typically used at UCLA to recruit individuals from the community (i.e., not UCLA subject-pool). Advertisements were posted on campus, primarily at the medical school and available public bulletin boards in the surrounding community, as well as posting on the Internet, especially using Craigslist and Facebook. One benefit of doing this design, is we are able to post Web-based ads nationally, so we recruited from a wider audience than just southern California.

**Figure 1:**
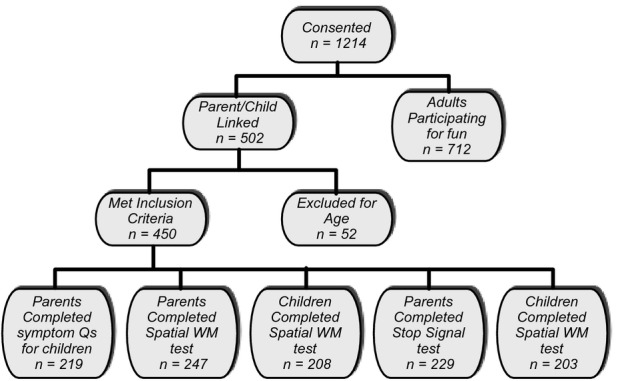
Consort diagram: a flow chart depicting the fate of all those who consented for this study. 1214 individuals consented. 502 parent–child pairs linked their accounts designating them a family. Of those, 450 individuals met inclusion criteria or 225 families.

Over 200 parent–child pairs did register linked family accounts and completed testing (see Fig. 1 for consort diagram). Families received a $50 gift card as compensation for participation after verification of email address, physical address, and age/gender of child who participated by our study coordinator as one way to help monitor study compliance. Demographic details are provided in Table 1. We maintained relaxed inclusion criteria in order to characterize a broad sample of community individuals who are willing to participate in Web-based testing. While not a true epidemiological sampling approach, our approach should minimize much systematic bias and allow us to estimate the true population of individuals participating via the web. In this respect, we also discouraged lying by minimizing reasons to do so (i.e., being more inclusive removes one reason to provide false answers). Exclusion criterion was self-report of an ADHD diagnosis.

**Table 1:** Demographic statistics shows age, gender, symptom sum, and responses to key medical history questions for the final sample analyzed here. Medical history shows the number of adult participants who self-reported past or present symptoms for themselves or their children

Description	Child	Parent
Mean age in years (SD)	13 (2.8)	37.3 (8.1)
Female	45%	67%
Attention symptom sum (SD)	9.24 (9.6)	
Medical history (# past/present)		
Epilepsy/unexplained loss of consciousness	0/0	0/0
Head injuries requiring hospital admission	2/2	1/2
Migraine	7/2	5/0
Asthma/bronchitis	12/10	1/1
Anxiety/depression	8/4	21/6
Other mental illness	4/0	4/0
Eating disorder	0/0	0/0
Unexplained weight loss	0/0	3/1

SD, standard deviation.

### Procedure

Parents created an account at http://BrainTest.org and then recruited their children. Both parent and child underwent informed consent/assent procedures on our website. They could read our “frequently asked questions” list or contact the study PI or support staff at anytime with questions. Following Simmons et al. (2011) recent paper on the potential for false positives in psychological research, we highlight every measure that was conducted and analyzed here. Children completed only the Friendship questionnaire (Baron-Cohen and Wheelwright 2003) and performed both a spatial working memory (SWM) task and the stop signal task (described below). Parents completed only those two same cognitive tests and a medical survey for themselves and their children as well as attention symptom scale and the Achenbach Childhood Behavioral Checklist (CBCL). The CBCL and Friendship questionnaire have not yet been analyzed.

### Measures

#### Medical questionnaire

The medical survey contained 22 items that broadly covered central nervous system conditions. The medical survey was completed by the parent for their own history as well as their child's. Allowable responses to the survey were for any of the four categories: “Child Presently,” “Child in History,” “Parent Presently,” and “Parent in History.”

#### Attention symptom scale

A scale was made for use by parents in the community to measure deficits in attention. It was adapted from the widely used 18 question adult self-report scale (ASRS), developed with the World Health Organization (Kessler et al. 2005). The ASRS, which is available on the Web, was developed as quick symptom screening tool in the community but does not confer a diagnosis of ADHD. Research does suggest, however, that those who score highly on this scale, do typically receive a diagnosis of ADHD using traditional diagnostic measures (Kessler et al. 2005). Responses were on a four-item Likert scale from “none/never” to “always.” Parents completed this scale about the attention behaviors of their children. The total sum and subscale sums for attention and motor questions were analyzed.

#### Spatial working memory

The SWM paradigm was developed using Flash (Adobe Systems, San Jose, CA) and designed to be identical in structure and design to one used in multiple center studies at UCLA (Cannon et al. 2002). Upon launch, a new window was opened and maximized on the participant's screen. After a brief practice to orient the participants and instruct on the proper response keys, participants performed four blocks of 16 trials. Data were collected in real-time on the client machine and sent back to the server at the end of each trial block using a 128-bit encrypted connection to avoid recording reaction times (RT) over the network. In this task, participants saw 1, 3, 5, or 7 dots presented on the screen in an abstract array for 2000 msec. After a delay of 3000 msec, a “probe” dot appeared for 3000 msec. and participants pressed one of two keys designated on the keyboard as to whether the probe dot was in the previously presented array or not. Working memory load (number of dots) was randomized across trials. Both RT and accuracy at each level of load were used as dependent variables. Prior to analysis, we did some initial data quality assurance, by excluding individuals who did not complete at least two blocks of trials and individuals who responded less than chance across multiple blocks. We also removed trials where participants responded in under 300 msec.

#### Stop signal task

The stop signal task has also been used extensively at UCLA (e.g., Cohen et al. 2010). We again designed a version in Flash with high face validity to one of the several versions used at UCLA. Participants saw either a left- or right-pointing arrow on the screen for 1000 msec and had to respond similarly using the arrow keys (inverted-t) on the keyboard. On 25% of the trials an auditory “beep” was presented and participants had to withhold their key press. The timing of the beep is adaptive and based on two alternating ladders (10 msec steps) in an attempt to find an optimized stopping time, while not allowing the participant to learn from a single ladder (Logan and Bundesen 2003). During instructions and practice, participants also performed a “speaker check” to ensure they could hear the auditory beep. The stop signal reaction time (SSRT) is typically the primary dependent variable, but also is highly sensitive to strategy effects (i.e., waiting, Logan and Bundesen 2003). In calculating SSRT, we found response patterns that suggested some participants may have been “waiting” despite our instructions, so we examined the RT on “go” trials, as well as the overall go accuracy and percent inhibition as more basic measures of inhibition and attention. Prior to analysis, we did some initial data quality assurance, by excluding individuals who did not complete at least two blocks of trials and individuals who responded less than chance across multiple blocks.

### Analytic approach

To establish construct validity of our Web-based adaptations of these widely used lab paradigms beyond face validity, we used a convergent validity approach frequently used in other forms of psychological testing (Messick 1989; McDonald 1999). We sought to determine: (a) if the pattern of association between the different tasks matches the pattern predicted by the relationship between the constructs they are assumed to tap into; (b) if the pattern of association between the different cognitive task and attention symptoms matches the pattern of association predicted by the relationship between the underlying constructs; and (c) use the dyadic nature of the data to determine if the relationship between the child's score on the different cognitive test and the parents score on the same test matches the degree to which neurocognitive endophenotypes are assumed to be heritable.

## Results

### Web sample characterization

**Figure 2:**
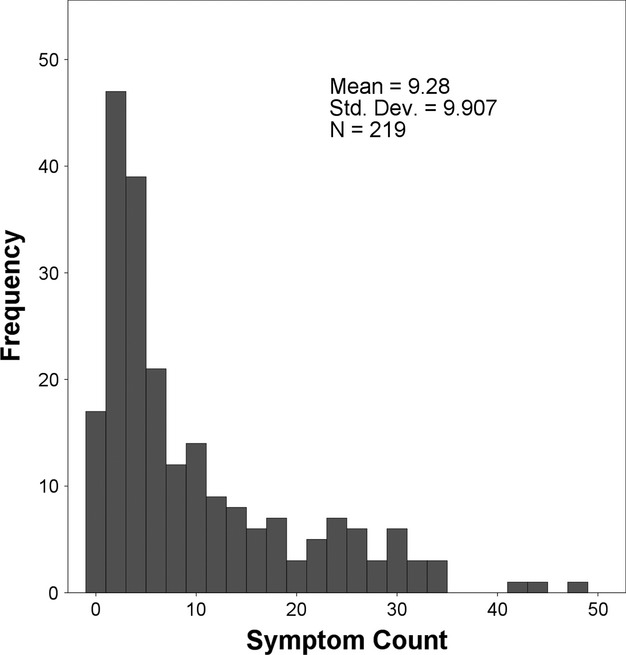
Distribution of symptoms shows the histogram for symptom sum in our adolescent Web sample. *Y*-axis shows frequency and *X*-axis shows sum total symptoms for each participant. Data report symptom scores for 219 adolescent participants as filled out by the participating parent.

Our initial goal was to characterize a large completely Web-recruited community sample without a diagnosis of ADHD on cognitive and symptom characteristics related to cognitive control. These data help provide normative data for Web-based cognitive test studies and begin to characterize those families who participate in Web-based studies. Table 2 highlights the cognitive test performance and the association with attention symptoms as well as the correlation in scores between parents and their offspring. Figure 2 shows the distribution of symptom sums for the children and adolescents in our sample.

**Table 2:** Performance characteristics on cognitive measures shows the raw scores for cognitive tests and statistics for comparison between family members

Description	Child score (mean)	Child score (SD)	Parent score (mean)	Parent score (SD)	Parent/child score difference (*P*-value)	Child's symptom score with child's score (correlation)	Child's score with parent's score (correlation)	Heritability (estimated as 2 × beta coefficient of the regression)
Spatial WM								
Load 1 Acc	92.8	9.54	94.7	5.95	0.08	0.04	0.06	0.12
Load 1 RT	899.6	315.71	909.2	274.74	0.87	0.27*	0.54**	1.08
Load 3 Acc	86.3	12.49	87.1	10.40	0.27	0.05	0.11	0.22
Load 3 RT	1031.8	354.63	1067.5	316.45	0.61	0.18*	0.66**	1.32
Load 5 Acc	78.9	13.66	79.5	12.69	0.62	0.02	0.36**	0.72
Load 5 RT	1079.9	357.06	1148.9	357.20	0.05	0.20**	0.65**	1.30
Load 7 Acc	78.8	13.55	78.6	12.18	0.79	−0.05	0.33**	0.66
Load 7 RT	1094.0	370.73	1155.9	355.11	0.10	0.20**	0.63**	1.26
Stop signal								
Go Trial RT	486.4	94.05	465.7	87.98	0.16	−0.05	0.62**	1.24
Go trial accuracy	96.6	5.00	97.3	3.69	<0.01**	−0.19**	0.27**	0.54
Percent inhibition	31.0	26.34	28.2	25.59	0.68	−0.23**	0.73**	1.46

* Significant at *P* < 0.05 and

** significant at *P* < 0.01.

Load, working memory load; Acc, accuracy; RT, reaction time; SD, standard deviation.

### Construct validation

**Figure 3:**
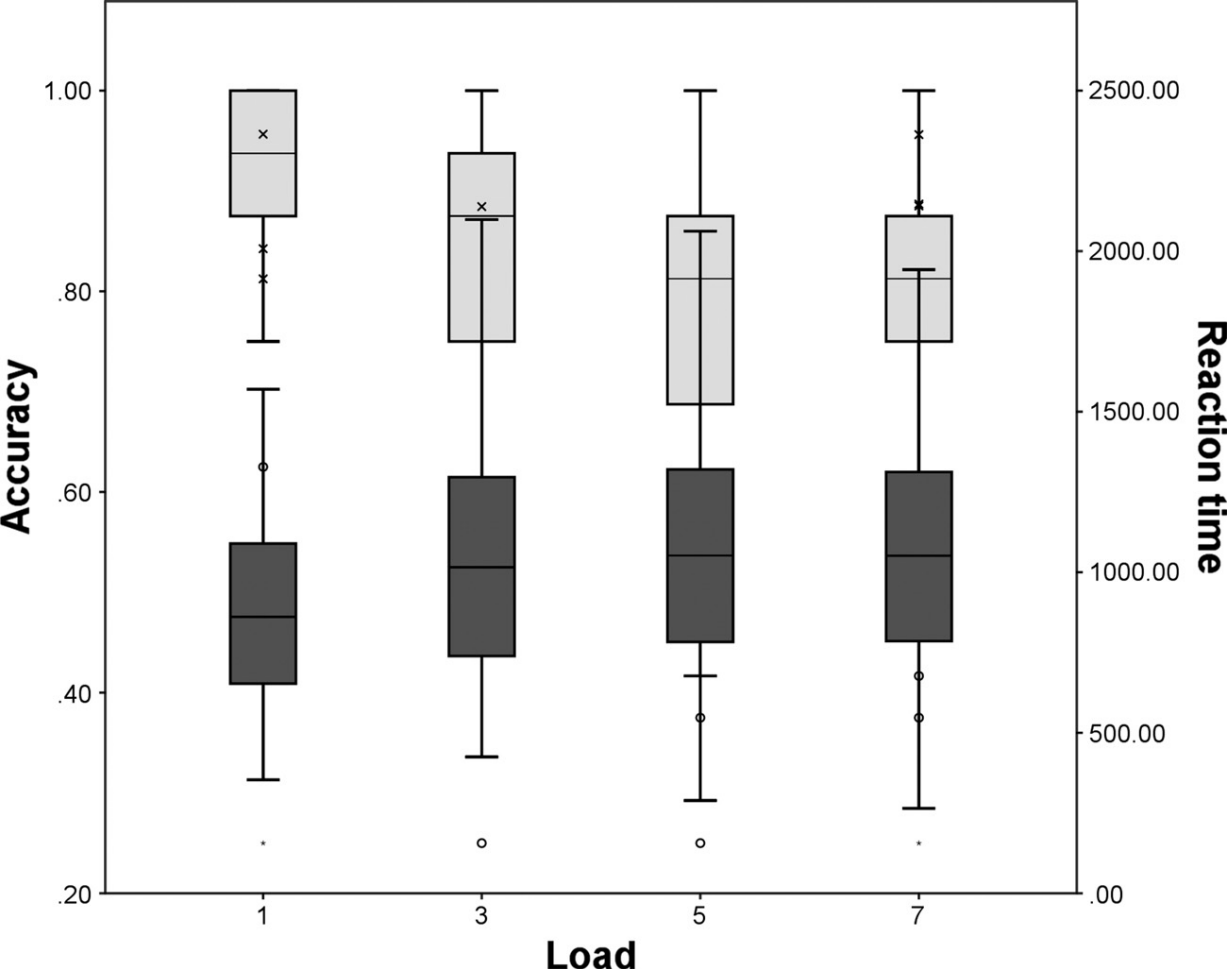
Behavioral performance shows box plots depicting the performance of the participants over the different load conditions is as expected: with increasing load the accuracy decreases and the reaction time increases. Left *Y*-axis shows Accuracy (acc) and right *Y*-axis shows reaction time (RT). *X*-axis shows the four different working memory loads (1, 3, 5, and 7 dots).

We examined the relationship between working memory and response inhibition measures to demonstrate an inverse relationship between the constructs in both children and parents (Table 3). The overall pattern of results is consistent across parents and their children: The expected correlation between the constructs of about 0.35 holds true for the relationship between response inhibition and working memory RT across all four load conditions. The data show internal validity with increased RT for higher working memory load and decreased accuracy (Fig. 3). Both tasks also showed strong reliability. The working memory task produced alphas that ranged from 0.81–0.85 across load-levels for Accuracy and 0.86–0.88 for RT using the first 10 trials. The stop signal task was even higher, (α = 0.98) for RT and (α = 0.96) for Accuracy based on first 100 trials.

**Table 3:** Correlations between tasks shows the relationship between performance on the spatial working memory task and the stop signal task for both parents and children

Description	Child			Parent		
	
	Mean RT	% Go	% Inhibition	Mean RT	% Go	% Inhibition
Load1 Acc	0.19*	0.28**	0.16*	−0.19**	0.01	−0.14
Load1 RT	−0.04	−0.20**	−0.32**	0	−0.13	−0.29**
Load 3 Acc	0.08	0.27*	0.18*	0.05	0.1	0.17*
Load 3 RT	−0.11	−0.18*	−0.38	−0.09	−0.1	−0.39**
Load 5 Acc	0.04	0.03	0.05	0.05	0.1	0.08
Load 5 RT	−0.08	−0.11	−0.33**	−0.05	−0.04	−0.32**
Load 7 Acc	0.01	0.12	0.04	0.1	0.06	0.18*
Load 7 RT	−0.08	−0.14	−0.36**	−0.11	−0.08	−0.39**

* Significant at *P* < 0.05 and

** significant at *P* < 0.01.

Load, working memory load; Acc, accuracy; RT, reaction time; SD, standard deviation.

### Cognitive control symptoms and behavior

We also examined summary statistics and correlations between symptoms and cognitive measures (Table 2), showing that the best measures are based on the criterion that they are associated to the predicted amount with attention symptoms and the RT across all four load conditions and percent inhibition, based on previous findings in the literature of relatively modest but significant correlations between inattention symptoms and working memory (e.g., Rogers et al. 2011), and consistent with neurocognitive profiles for ADHD (Walshaw et al. 2010).

The correlations between the child and the parent measures on working memory and response inhibition variables are high (e.g., *r* = 0.73; Percent inhibition), suggesting our tasks may have significant heritability (e.g., similar to the heritability for Intelligence = 0.75, Nessier et al. 1996), and thus would be appropriate for use in genetic association studies or useful as endophenotypes in psychiatric research (Gottesman and Gould 2003). Examining correlations and narrow-sense heritability (double the slope of the regression) between parent and offspring can reveal the ceiling of potential heritability, but does not properly control for epistasis or environment effects (Lynch and Walsh 1998).

In order to address the confound of potential shared variance between parent and child due to shared computing equipment and testing environment we conducted a leverage analysis (see Table 5). We determined how much of the parents and child's RT would have to be explained by shared computing equipment and other testing environmental factors by assuming that it is possible to decompose the observed covariance between parent and child into two components: One due to the familial connection between parent and child, and one that is due to shared testing environment. In this model it is possible to determine how large a proportion of the observed covariance would have to be due to the shared environment to make the rest of the covariance – assumed to be due to actual association between parent and child not significantly different from zero. The estimates for the variability in parent and child scores due to this shared environment component range from a standard deviation of 33 msec (Go Trial RT) to a standard deviation of 154 msec (WM Load 5 RT). While this is not conclusive, and the effect of shared computing equipment and testing environment needs to be tested directly, evidence from the literature on stimulus timing (e.g., Li et al. 2010) suggests that differences typically observed among computers, including architecture and peripherals, like keyboards and mouse devices, are likely not enough to completely account for our heritability results.

Finally, we examined correlations between parent and child cognitive performance across three different levels of attention symptoms. Previous work has suggested nonlinear relationships between symptoms and behavior (Lubke et al. 2007). Children's scores on the attention survey were broken into three equal-sized groups. The first group had self-reported symptoms that summed to less than three (“low” group). The second group had self-reported symptoms that summed to between three and ten (“medium” group). The final group had self-reported symptoms that summed to more than ten (“high” group). We examined the significance of only our three most promising indicators from previous analyses: Working Memory Load 3 and Load 5 Reaction Time and Stop Signal Percent Inhibition. Table 4 presents the correlations by bin and the *P*-values associated with those correlations. These data suggest that while symptoms and behavior represent quantitative traits along a continuum, the relationship changes, which may suggest different latent classes (Lubke et al. 2009).

**Table 4:** Correlations in symptom bins shows the correlations between parent and children across three different symptom groups for spatial working memory and the stop signal

Parent–child correlations × symptom group	Correlations			*P*-values		
	
	Low Symp (<3) *N* = 64	Med Symp (3–10) *N* = 76	High Symp (>10) *N* = 79	bin1 v. bin2	bin1 v. bin3	bin2 v. bin3
WM RT_Load3	0.636	0.833	0.475	**0.017**	0.226	**<0.001**
WM RT_Load5	0.578	0.831	0.492	**0.004**	0.535	**0.001**
Percent_Inhibition	0.767	0.718	0.473	0.55	**0.01**	0.049

Load, working memory load; RT, reaction time.

Bold values highlight significant findings.

**Table 5:** Leverage analysis of potential errors due to parent and child using the same computing equipment

	Estimated standard deviation of the correlation coefficients explainable by a 20 msec noise	Lower bound correlation estimate (−4 SD)	Heritability based on the lower bound correlation estimate	Shared variance due to computing equipment necessary to make correlation nonsignificant
Load 1RT	0.0047	0.54**	1.08	SD = 89
Load 3RT	0.0036	0.66**	1.32	SD = 148
Load 5 RT	0.0031	0.65**	1.30	SD = 154
Load 7 RT	0.0032	0.63	1.26	SD = 150
Go Trial RT	0.0145	0.61	1.21	SD = 33

** Significant at *P* < 0.01.

Load, working memory load; RT, reaction time.

## Discussion

Understanding the neurobiology of behavioral constructs like cognitive control will require testing participants using unsupervised and parallel approaches. We present novel findings on symptom prevalence in the web community of adolescents, an interaction between symptoms and cognitive test performance, and strong suggestion of significant heritability of measures frequently used to examine cognitive control. Running hundreds or thousands of participants in lab-based studies is extremely inefficient and practically impossible to execute in a timely manner. Although studies have shown scores on lab-based measures to be highly correlated with those online, there remains skepticism about this approach. In our study, we used typical construct validity tests done for new psychological measures to support our findings. Given this, we suggest consistent use of the Web for cognitive assessment will help overcome continued inertial bias for lab-based cognitive testing and be instrumental in uncovering the genetic bases of behavior.

We sought to characterize a community sample without a diagnosis of ADHD recruited entirely using the web. As such, this is not a “super control” sample (attention symptom sum ranges from 0 to 47). This increases ecological validity and provides additional power for correlations as the data encompasses a large range of scores. It does, however, make it difficult to compare the results directly to prior studies with either clinical patients or typical lab-based control populations, but does represent an important characterization of the symptoms in the community-at-large that can begin to establish Web-normative scores. Our finding of symptom scores across a large range, in children and adolescents without a self-reported diagnosis of ADHD is important and novel for a Web-based community. Recent epidemiological reports from the Centers for Disease Control suggest the community prevalence of a diagnosis of ADHD is over 8% (http://www.cdc.gov). Few studies, however, have looked broadly at symptoms that exist in the community. Our attention symptom finding supports reports that ADHD-related symptoms are dimensional (Lubke et al. 2009), and should be treated as quantitatively distributed traits in the population. Yet, similar to Lubke et al. (2009), we do find that cognitive test performance changes as function of symptom level, which may suggest different latent classes. These data may improve the ability to track the underlying genetic contribution of these symptoms.

Our findings of high correlations between parent and offspring scores on our cognitive control measures suggest high heritability of these constructs, an important step in investigating genetic associations. Typically, examining heritability is difficult for new computerized measures, as recruiting and testing families in a large enough sample to measure heritability is not feasible. Further, with iterative development of new measures, it becomes more challenging for phenotypes to be adequately validated with respect to genetic studies. Studying a single parent and offspring allows us to compute narrow-sense heritability or what some have called biometric heritability (Lynch and Walsh 1998). These numbers provide a ceiling for additive genetic influences without taking into account shared environment or pure environment factors or epistasis. Our calculations of narrow-sense heritability suggest high heritability but also unsurprisingly that these unmeasured sources of variance do play a role in working memory and response inhibition. They also suggest that some phenotypic indicators may not be useful in genetic association experiments going forward, as they display very low narrow-sense heritability (e.g., Working Memory load accuracy at low loads). These findings suggest our approach is feasible and extremely efficient for examining these questions, but larger pedigree-type data would be ideal for answering these questions. While further research needs to be done to fully address the technical considerations of conducting heritability research remotely using varying equipment, ideally through direct recording of these variables and ensuring family members use different computers, there is evidence in the literature (e.g., Li et al. 2010) suggesting that some of the confounds in computer architectures and peripheral equipment are likely not enough to completely account for our heritability findings. As such, these results may be useful in the future in estimating the size of the effect of hardware/software noise as more detailed data about these sources of noise are studied.

This study also supports our hypothesis about the validity of web assessment of cognitive control. These tests show excellent face validity based on well-established paradigms and demonstrate evidence of construct validity. We also provide additional evidence in showing that the association between both RT and inhibition with the attention symptoms is consistent with the literature (Walshaw et al. 2010). This approach is the same used in other domains of psychological testing (Block et al. 1974; Reynolds and Koback 1995), and while we show somewhat more moderate effect sizes than these psychometrically built instruments, our procedures are identical to other computerized test development. Although typically not seen with new computerized cognitive test development, Gur and colleagues did use a similar approach to demonstrate validity of a larger cognitive test battery (Gur et al. 2010). This is in contrast to previous studies, which have pursued equivalence testing metrics to theoretically ensure tests are identical across testing platforms. Our approach focuses on construct validation using tasks with extremely high face validity. Very few new lab-based variations of cognitive paradigms undergo equivalence testing. Web-based tests that are demonstrated to measure latent constructs of interest should be adequate in assessing cognitive control behavior.

With the ubiquity of the web in our daily lives, it follows that cognitive testing should use web technology*,* especially as the knee-jerk theoretical biases have been consistently shown to be surmountable. While the sample biases typically associated with Internet-research have been shown to be less problematic in direct examination (Gosling et al. 2004; Haworth et al. 2007), there are typically *more* demographically varied samples found online, where any study can recruit from millions of potential participants. This is not to suggest that the Web does not have sample biases, but as these studies have shown, the biases are not different from those typically seen in lab-based psychological studies where recruitment is almost never truly random. The benefit with using the Web, is that you can sample from a much larger pool than will be available in a typical lab study (i.e., every demographic category can be found in greater number on the Web than within participation distance of any single institution).

The primary concern about web testing, however, has been response bias. There is a large body of evidence showing high correlations (>0.7–0.8) between web and lab assessment in the same individuals (Buchanan and Smith 1999; Krantz and Dalal 2000; Gosling et al. 2004; Bedwell and Donnelly 2005; Haworth et al. 2007; Silverstein et al. 2007; Younes et al. 2007; Germine et al. 2012). Buchanan (2003) argues that solely because an assessment was adapted from lab- to Web-based format one cannot assume the newer version has the same psychometric properties. While true, to conclude that this means that web versions are not useful is premature, rather a web test should be considered a new measure, with its own psychometric properties and norms. The construct validation approach used here and by others previously (Krantz and Dalal 2000; McGraw et al. 2000; Silverstein et al. 2007) builds upon the growing evidence base for the valid adoption of Web-based assessment of cognitive control.

Web testing provides novel experimental design opportunities for examining the underlying genetic substrates of behavior. While methodologies for examining the genetic associations have improved dramatically in the last several years, efforts aimed at clarifying phenotypic expression have lagged, especially in neuropsychiatry (Sabb et al. 2009). The common misconceptions about the pitfalls of web testing have shown to be no worse than pitfalls seen in laboratory testing, but efficiency and cost-effectiveness are unparalleled with web testing. We demonstrated the power of this approach by efficiently recruiting a large family sample, which revealed the prevalence of subclinical attention symptoms in the web community. We also demonstrated an interaction between cognitive test performance and symptom level that may have implications for more broadly understanding ADHD. Finally, our data suggests a “ceiling” for the heritability of these Web-based cognitive control measures. This may aid cognitive control phenotype selection for genetic analyses going forward, as several indicators had particularly low ceilings and could be avoided. More broad adoption of the web for testing is needed to demonstrate test–retest reliability in web scores and establishment of Web-based norms. If successful, this approach could greatly increase our ability to understand the underlying neurobiology of behavioral constructs that are core components of neuropsychiatric diseases.
